# Presence of human papillomavirus DNA in breast cancer: a Spanish case-control study

**DOI:** 10.1186/s12885-017-3308-3

**Published:** 2017-05-08

**Authors:** Silvia Delgado-García, Juan-Carlos Martínez-Escoriza, Alfonso Alba, Tina-Aurora Martín-Bayón, Hortensia Ballester-Galiana, Gloria Peiró, Pablo Caballero, Jose Ponce-Lorenzo

**Affiliations:** 1 Department of Obstetrics and Gynecology, University General Hospital of Alicante, c/ Pintor Baeza, 11, 03010 Alicante, Spain; 2 Department of Genetics, Institute of Cellular and Molecular Studies, Lugo, Spain; 3 Department of Pathology, University General Hospital of Alicante, Institute of Sanitary and Biomedical Research of Alicante (ISABIAL), Alicante, Spain; 4 Department of Community Nursing, Preventive Medicine and Public Health and History of Science, University of Alicante, Alicante, Spain; 5 Department of Medical Oncology, University General Hospital of Alicante, Alicante, Spain

**Keywords:** Breast cancer, Human papillomavirus, Prevalence, PCR

## Abstract

**Background:**

Breast cancer is one of the most important neoplasia among women. It was recently suggested that biological agents could be the etiological cause, particularly Human Papilloma Virus (HPV). The aim of this study was to explore the presence of HPV DNA in a case-control study.

**Methods:**

We performed our study including 251 cases (breast cancer) and 186 controls (benign breast tumors), using three different molecular techniques with PCR (GP5/GP6, CLART® and DIRECT FLOW CHIP®).

**Results:**

HPV DNA was evidenced in 51.8% of the cases and in 26.3% of the controls (*p* < 0.001). HPV-16 was the most prevalent serotype. The odds ratio (OR) of HPV within a multivariate model, taking into account age and breastfeeding, was 4.034.

**Conclusions:**

Our study, with methodological rigour and a sample size not previously found in the literature, demonstrate a significant presence of HPV DNA in breast cancer samples. A possible causal relationship, or mediation or not as a cofactor, remains to be established by future studies.

## Background

Breast cancer is the most commonly diagnosed malignancy in women [1–3]. It is estimated that 1.7 million new cases were diagnosed in 2012, representing 11.9% of all cancers diagnosed worldwide in both genders, and 25% of those diagnosed in women [3, 4]. Breast cancer is also the most common malignancy in Spanish women, representing 29% of all female malignancies. Most of the cases are diagnosed in patients between 45 and 65 years of age [5].

Several risk factors have been cited in the literature, including patient age, gender, hormone therapy, the number of offspring, breastfeeding or different eating habits. However, there are other less well known factors that might also play an oncogenic role. Viruses are an example in this respect [6]. A number of viruses have been identified to date in breast cancer tissues. The three main viruses are Epstein Barr virus (EBV), mouse mammary tumor virus (MMTV) and human papillomavirus (HPV) [7–11]. All of them share a common feature in that they can induce the initiation and progression of cancer. Several studies [8, 9, 12–19] have attempted to determine whether viruses in breast tissue are a casual presence (i.e., acting as “passengers”) or they play an important role in carcinogenesis. The fact is that with the exception of MMTV, the rest of the viruses described in breast cancer have already been identified in other malignancies. The current published data on HPV and breast cancer are very contradictory, since the reported prevalence of HPV ranges from 0% [20–29] to 86.21% in breast cancer tissue samples [8, 10, 30–35]. Furthermore, the studies are very heterogeneous in terms of the methodology employed. A example of this is that, most of the reviewed studies involve case studies without controls. A few use case-control protocols, which offer greater methodological soundness, while only a handful evaluate statistically significant differences [9, 31, 36–43]. Moreover, the only study conducting logistic regression is that published by Sigaroodi et al. [40], though it involves a very wide confidence interval (1.5–130) and odds ratio [OR] = 14, which is questionable in statistical terms.

In view of the above, the investigation of viruses as breast cancer promoting factors remains subject to great controversy. The present study was designed to help clarify this issue. Specifically, we aimed to confirm the presence of HPV in a series of samples obtained from breast surgeries at the University General Hospital of Alicante (Spain), estimating the strength of the association (via [OR]) between the presence of HPV in benign breast disease and breast cancer.

## Methods

A case-control study, based on a case-control ratio of 1:1, was performed to evaluate the presence of HPV infection in a subset of 250 embedded breast cancer tissues, as cases, and 250 embedded benign breast tissues, as controls. The estimated exposure rate (presence of HPV) was 25% and 14% in the cases and controls, respectively, with a confidence level of 95% and a statistical power of 85% in detecting OR >2 (computed pooling proportions of reviews or meta-analyses published until 2012) [8, 30, 32]. The samples were selected consecutively and retrospectively from the year 2012 until the calculated sample size (n) was reached. The following inclusion criteria were established: women subjected to surgical treatment due to infiltrating breast cancer and/or carcinoma in situ (cases) or benign breast disease (controls) (period 2006–2012); patients over 18 years of age; surgical specimens embedded in paraffin (stored in the tumor Biobank of our institution), in adequate conditions and sufficient amount of tissue for the purposes of the study; and the obtainment of written informed consent. The following exclusion criteria were established: males and a lack of the minimum required quality controls in the analyzed DNA samples. An ad-hoc case report form was created to record demographic, histopathological and virological information. Data were anonymized in compliance with the protection of personal data code.

### Immunohistochemical and in SITU hybridization analysis

After surgical excision (either mastectomy or tumorectomy), specimens were fixed in 10% formalin solution and subsequently embedded in paraffin. For the histological study, sections measuring 4 μm in thickness were obtained and stained with hematoxylin-eosin. The expressions of estrogen receptor (ER), progesterone receptor (PgR), human epidermal growth factor receptor (HER2) and Ki-67 were determined by immunohistochemistry (IHC) using standard techniques, with commercial antibodies and conditions following the instructions of the manufacturer on an automated basis (Techmate-500). The following antibodies were used: ER (Dako, clone 1D5, dilution 1:50), PgR (Dako, clone PgR 636, dilution 1:50), Ki-67 (Dako, clone MIB-1, dilution 1:100) and HercepTest® (Dako). The study of the ER and PR expression levels was made evaluating the percentage of stained tumor cell nuclei and the intensity of staining according to the guidelines of the American Society of Clinical Oncology (2010) [44] and of the American College of Pathologists. Positive status was considered for >1% ER or PR. HER2 status in turn was determined according to the recommendations of the American Society of Clinical Oncology (2007) and guidelines of the American College of Pathologists [45]. Immunohistochemical positive was defined as staining 3+ (uniform, membrane staining intensity >10% of the infiltrating tumor cells), while negative was defined as staining 0 or 1+. *ERBB2* gene status was confirmed by fluorescence in situ hybridization (FISH) (Dako pharmaDx™) or chromogenic in situ hybridization (CISH) (Spot light™; Zymed) in equivocal cases (2+ and <10% 3+ cells). Ki67 was semiquantitatively assessed in at least three high-magnification fields [×400] including hot-spots areas, and classified as low (<14%) versus high (>14%) (nuclei) [44, 45].

### Viral DNA sequences extraction

The search for viral DNA was carried out at the Instituto de Estudios Celulares y Moleculares (Lugo, Spain), due to its well demonstrated experience in molecular and genetic studies. Sections measuring 10 μm in thickness were obtained from the tumor area of the paraffin block for the identification of viral DNA. In order to avoid cross-contamination between samples, special care was taken in handling and sectioning the samples. The following procedure was carried out:

1. Deparaffinization: Four paraffin-embedded tissue sections were placed in a 1.5-ml tube, followed by the addition of 1 ml of xylene and vortexing for 10 s. After incubation at room temperature during 10 min, centrifugation was carried out at 13,000 rpm for 5 min. The supernatant was discarded and 1 ml of absolute ethanol was added, followed by centrifugation at 13,000 rpm for 2 min. The supernatant was then again discarded. This ethanol washing step was repeated one more time. Lastly, the sample was incubated at 56 °C during 15 min to eliminate the traces of ethanol.
2. DNA extraction: After completion of the deparaffinization process we added 500 μl of lysis buffer (10 mM Tris pH 8, 100 mM NaCl, 25 mM EDTA, 0.5% sodium dodecylsulfate [SDS]) and 10 μl of proteinase K (20 mg/ml), followed by vortexing and incubation in a shaking water bath at 56 °C overnight. Proteinase K was inactivated by incubation at 95 °C during 10 min. An equivalent volume of chloroform: isoamyl alcohol (24:1 *v*/v) was added, shaking gently by inverting the tube and then centrifuging at 10,000 rpm for 10 min. The upper aqueous phase was transferred to a new microcentrifugation tube, and 0.2 volumes of ammonium acetate 10 M were added. The DNA was precipitated by adding two volumes of absolute ethanol, followed by vortexing for 5 s, incubation at −20 °C during 30 min and centrifugation at 10,000 rpm for 20 min. The supernatant was discarded and the precipitate was washed with 500 μl of cold 70% ethanol, followed by centrifugation for 5 min at 10,000 rpm. After discarding the supernatant again, the precipitate was dried at room temperature during 20 min. The DNA was finally resuspended in 50 μl of Tris-EDTA solution.
3. Amount and quality of DNA: All DNA samples were analyzed using a *Nanodrop 1000* kit allowing calculation of the concentration of DNA and the A260/A280 and A260/A230 ratios, which indicate the purity of the molecule.
4. DNA amplification capacity: The integrity of the extracted DNA was evaluated by polymerase chain reaction (PCR) amplification of a fragment of the methylenetetrahydrofolate reductase (MTHFR) gene.
5. Detection and genotyping of HPV: The samples were subjected to three different HPV detection methods:
- Amplification of the virus using the GP5+/GP6+ consensus primers: The presence of HPV DNA was evaluated by PCR using the GP5+/GP6+ primers (150 bp), which act as consensus primers for the HPV L1 gene. The PCR reaction was carried out with 5 μl of DNA in a total reaction volume of 50 μl containing 25 μl of DreamTaq Green PCR Master Mix 2X (ThermoFisher Scientific), 1 μM of each primer, 0.2 mM of DNTPs and 2 mM of MgCl_2_. Amplification was performed with initial activation of the enzyme at 95 °C during two minutes, followed by 45 cycles under the following conditions: 30 s at 95 °C, two minutes at 40 °C and 1.5 min at 72 °C, with a final elongation step at 72 °C during 5 min. The PCR products were visualized in 2% agarose gel with ethidium bromide staining using electrophoresis.
- CLART® HPV2 amplification kit (Genomica): This kit detects the presence of the 35 HPV viruses: 6, 11, 16, 18, 26, 31, 33, 35, 39, 40, 42, 43, 44, 45, 51, 52, 53, 54, 56, 58, 59, 61, 62, 66, 68, 70, 71, 72, 73, 81, 82, 83, 84, 85 and 89. Detection is carried out through amplification of a fragment of about 450 bp within the L1 region of the virus. Five μl of DNA of each sample were subjected to PCR assay using the CLART HPV2 amplification kit (Genomica): one cycle at 95 °C for 5 min, 40 cycles at 94 °C for 30 s/55 °C for 60 s / 72 °C for 90 s, and one cycle at 72 °C for 8 min. The PCR products were denaturalized at 95 °C during 10 min and placed in a container with ice. Hybridization was performed using 10 μl of the denaturalized PCR product in the CLART microarray, followed by examination according to the instructions of the manufacturer.
- HPV Direct Flow CHIP kit (Master Diagnostica): The technique is based on amplification of the viral DNA followed by membrane flow-through reverse dot blot hybridization of the amplified products. Types of HPV detected: High oncogenic risk (16, 18, 26, 31, 33, 35, 39, 45, 51, 52, 53, 56, 58, 59, 66, 68, 73 and 82) and low oncogenic risk (6, 11, 40, 42, 43, 44, 54, 55, 61, 62, 67, 69, 70, 71, 72, 81, 84 and 89 (CP6108)). Six μl of purified DNA of each sample were amplified by PCR under the following conditions: one cycle at 98 °C for 5 min, 5 cycles at 98 °C for 5 s / 42 °C for 5 s / 72 °C for 10 s, 45 cycles at 98 °C for 5 s / 60 °C for 5 s / 72 °C for 10 s, and one cycle at 72 °C for one minute. The samples were kept in refrigerated tubes (8–10 °C) until processing. The PCR products were denaturalized by heating to 95 °C for 5 min (in a thermocycler) and then quickly cooled in ice for two minutes.

Hybridization and interpretation of the results were carried out following the instructions of the kit manufacturer. All samples were analyzed using the three techniques above mentioned to increase test sensitivity. A positive result was defined when at least two of the three methods detected the presence of HPV. If the results proved questionable, or in the event of insufficient material, the sample was discarded to the effects of analysis. Likewise, all the samples passed the cellular DNA test (internal control), to avoid possible false-negative results. As negative control we included “HPV free DNA” and as positive control a HPV plasmid mixture with all target types. Contradictory results were obtained in only 6 samples, and these were therefore considered lost.

The following variables were recorded: patient age, personal breast cancer history, smoker, number of children, breastfeeding, age at menopause, history of cervical disease, adjuvant therapy, histopathological of the tumor (tumor size, grade, stage, number of positive lymph nodes, local/distant metastasis), immunohistochemical characteristics (ER, PgR, HER2, Ki67), and the detected HPV serotype.

### Data analysis

A descriptive analysis was made of all the study variables. In order to analyze the homogeneity of the two groups (cases and controls) with respect to those variables which the literature describes as being associated to breast cancer, a calculation was made of the means of the quantitative variables for both groups and comparisons were established using the Student *t*-test. In the case of the qualitative variables, cross tables were generated, and associations were analyzed using the Chi-Squared test. In addition, any variables (qualitative or quantitative) found to be non-homogeneous in the cases and controls were taken into account when explaining the lack of homogeneity for the other variables. To this effect, we calculated the strength of the association between the cases and controls with the variable in question, in the presence of those variables which had already demonstrated a lack of homogeneity in both groups. This process was carried out using a binary logistic regression model. In order to establish the association between HPV exposure and breast cancer, we generated a cross tables between the two variables, estimating the magnitude of the association based on calculation of the raw odds ratio (OR) for the development of breast cancer. In addition, we calculated the adjusted OR by binary logistic regression, with the corresponding 95% confidence interval (CI). A *p*-value <0.05 was considered statistically significant. Statistical analysis was carried out using the software package SPSS version 20.

## Results

The final study included 437 samples: 251 cases (57.4%) and 186 controls (42.6%). (Table 1).

**Table 1:** Clinical characteristics of breast cancer tissues (cases) and breast benign diseases (controls)

	Cases		Controls	
	*N*	Mean (CI 95%)	*N*	Mean (CI 95%)
Age**	251	56.32 (54.76–57.88)	186	40.08 (38.3–41.86)
Number of children	250	1.92 (1.76–2.09)	185	1.3 (1.11–1.5)
Breastfeeding duration (months)	191	8.65 (7.16–10.15)	115	9.42 (6.92–11.92)
Age at menopause	140	48.89 (48.16–49.61)	36	48 (46.47–49.53)
Tumor size (mm)	249	29.49 (26.99–31.99)	98	31.83 (29.31–34.34)

*CI 95%* Confidence Interval 95%, ** Statistic Signification <0.01

The mean age of the cases (*n* = 251) was 56.3 years, versus 40.1 years for the controls (*n* = 186) ((*p* < 0.001). Of note, the two groups are not homogeneous. A statistically significant difference of 16 years was observed between the cases and controls (Tables 1 and 2). This is justified on the basis of the natural courses of breast cancer and benign disease, and the curves corresponding to breast cancer and benign disease by ages described by the World Health Organization (WHO) [4] are analogous to those of our own study. Clearly, for ethical reasons, we cannot obtain healthy tissues on a random basis from health women without breast disease. Selection bias results if the groups are not homogeneous. However, by using statistical tools such as binary logistic regression analysis, we can control this bias referring to the lack of homogeneity in terms of variables which presumably may be related to cancer. Furthermore, this statistical tool allows us to calculate odds ratios for each variable. After justification of the variable age, we compared the rest of the variables of the cases and controls (Table 2), and these were found to be homogeneous after logistic regression adjustment to age. This allows us to establish statistical comparisons of our primary variable, which is the presence or not of viral DNA.

**Table 2:** Frequency of cases and controls by clinicopathological factors

		Number	Cases *n* (%)	Controls *n* (%)	*p*-value (*p*-value adjusting for age)	OR (CI 95%)	AOR by Age (CI 95%)
Age (years)	< 40	109	19 (7.6)	90 (48.4)	<0.001	1	−
	40–49	119	60 (23.9)	59 (31.7)		4.8 (2.6–8.9)**	−
	50–59	97	72 (28.7)	25 (13.4)		13.6 (7.0–26.7)**	−
	≥ 60	112	100 (39.8)	12 (6.5)		39.5(18.2–85.8)**	−
Smoker	Yes	196	93 (37.7)	103 (56.3)	<0.001 (0.446)	0.5 (0.3–0.7)**	0.8(0.5–1.2)
	No	234	154 (62.3)	80 (43.7)		1	1
Personal BC history	Yes	13	12 (4.8)	1 (0.5)	0.009 (0.086)	9.3 (1.2–72.1)**	7.0(0.8–64.7)
	No	424	239 (95.2)	185 (99.5)		1	1
Number of children	1+	326	205 (81.7)	121 (65.1)	<0.001 (0.465)	2.4 (1.5–3.7)**	1.0 (0.6–1.8)
	None	111	46 (18.3)	65 (34.9)		1	1
Breastfeeding (months)	12+	81	52 (27.2)	29 (25.2)	0.789 (0.139)	1.1 (0.6–1.9)	0.8 (0.4–1.5)
	0–11	170	139 (72.8)	86 (74.8)		1	1
Menopausial status	Yes	188	152 (62.0)	36 (19.7)	<0.001 (0.238)	6.7 (4.2–10.4)**	0.7 (0.3–1.6)
	No	240	93 (38.0)	147 (80.3)		1	1
Age at menopause (years)	≥ 54	25	24 (15.8)	1(2.8)	0.012 (0.179)	18 (1.9–173)**	7.5 (0.7–82.6)
	42–53	149	120 (78.9)	29 (80.6)*		3.1 (0.9–9.6)*	1.8 (0.5–6.9)
	≤ 41	14	8(5.3)	6 (16.7)		1	1
History of cervical diseases	No	302	156 (95.7)	146 (91.8)	0.171 (0.288)	1.9 (0.8–5.1)	1.7 (0.6–4.8)
	Yes	20	7 (4.3)	13 (8.2)		1	1

*OR* Odd Ratio, *AOR* Adjusted Odd Ratio, *BC* Breast Cancer, *CI 95%* Confidence Interval 95%, * Statistic signification <0.05, ** Statistic Signification <0.01

Data obtained to determine the presence of HPV in breast cancer tissue samples and establish the comparison with the samples corresponding to benign breast tissue, were analyzed using the Chi-Squared test. Using this test, the HPV exposure rate among the cases was significantly higher (51.8%) than the HPV exposure rate in the controls (26.3%) (*p* < 0.001) (Table 3).

**Table 3:** Frequency of HPV-positive by cases and controls

		Cases (*n* = 251)		Controls (*n* = 186)		OR (CI 95%)
		N	%	N	%	
HPV	HPV+	130	51.8%	49	26.3%	3.0 **
	HPV-	121	48.2%	137	73.7%	(2.0–4.5)

** Statistic Signification <0.01, *CI 95%* Confidence Interval 95%

The raw OR was 3.0 (CI 95%: 2.0–4.5). On applying the binary logistic regression model to control for confounding variables, the OR assigned to HPV was seen to be 4.034 (CI 95%: 2.213–7.352) (Table 4), which means a higher risk of suffering cancer in the presence of HPV, taking into account patient age and breastfeeding. The rest of the confounding variables showed no significance in the binary logistic regression model (all *p* = non significant).

**Table 4:** Binary logistic regression model to control for confounding variables in a case-control study

	Coef. B	Sig.	OR.	CI. 95%
HPV	1.395	<0.001	4.034	2.213–7.352
Age	0.11	<0.001	1.116	1.084–1.15
Breastfeeding	−0.032	0.022	0.969	0.943–0.996
Constant	−5.274	<0.001	0.005	

Coef. B. Value of the coefficient in the logistic regression model. *Sig* Statistic signification, *OR* Odd Ratio, *CI 95%* Confidence Interval 95%

The mean tumor size in HPV-positive tumors was larger (30.53 mm) than in those HPV-negative (28.37 mm), though the difference failed to reach statistical significance (*p* = 0.395). None of the analyzed histopathological variables showed a statistically significant association with the presence of HPV (Table 5, *at the end*).

**Table 5:** Frequency of HPV-positive cases (Breast cancer) by clinicopathological factors

		Number	HPV+		HPV-		P*	OR (CI 95%)
			*n*	%	*n*	%		
Lymph vascular invasion	Yes	43	23	17.7%	20	16.5%	0.868	1.1 (0.6–2.1)
	No	208	107	82.3%	101	83.5%		1
Lymph node metastasis	Yes	104	52	40.0%	52	43.0%	0.701	0.9 (0.5–1.5)
	No	147	78	60.0%	69	57.0%		1
Metastasis	Yes	10	6	4.6%	4	3.3%	0.751	1.4 (0.4–5.1)
	No	241	124	95.4%	117	96.7%		1
Neoadjuvant therapy	Yes	33	16	12.3%	17	14.0%	0.712	0.9 (0.4–1.8)
	No	218	114	87.7%	104	86.0%		1
Stage	0	25	14	10.9%	11	9.3%	0.724	1
	IA	51	30	23.4%	21	17.8%		1.1 (0.4–2.9)
	IB	1	1	0.8%	0	0.0%		…
	IIA	78	35	27.3%	43	36.4%		0.6 (0.3–1.6)
	IIB	45	25	19.5%	20	16.9%		1.0 (0.4–2.6)
	IIIA	32	15	11.7%	17	14.4%		0.7 (0.2–2.0)
	IIIC	3	2	1.6%	1	0.8%		1.6 (0.1–20.0)
	IV	11	6	4.7%	5	4.2%		0.9 (0.2–3.9)
ER	0	39	18	14.1%	21	17.5%	0.407	2.6 (0.2–27.0)
	1–19%	4	1	0.8%	3	2.5%		1
	≥20%	205	109	85.2%	96	80.0%		3.4 (0.3–33.1)
PgR	0	51	26	20.3%	25	20.8%	0.581	0.7 (0.3–1.7)
	1–19%	37	22	17.2%	15	12.5%		1
	≥20%	160	80	62.5%	80	66.7%		0.7 (0.3–1.4)
HER2	+	43	27	21.3%	16	13.8%	0.134	1.7 (0.9–3.3)
	−	200	100	78.7%	100	86.2%		1
Ki-67	< 14	84	37	28.9%	47	39.5%	0.083	1
	14–19*	71	44	34.4%	27	22.7%		2.1 (1.1–3.9)
	≥ 20	91	47	36.7%	45	37.8%		1.4 (0.7–2.5)
Immunohisto-chemical subtypes	LUMINAL A	88	37	29.4%	51	44.0%	0.055	1.2 (0.4–4.0)
	LUMINAL B/HER2-	83	50	39.7%	33	28.4%		2.1 (0.6–7.1)
	LUMINAL B/HER2+	35	23	18.3%	12	10.3%		2.7 (0.7–10.4)
	HER2+	12	5	4.0%	7	6.0%		1
	TRIPLE NEGATIVE	24	11	8.7%	13	11.2%		1.2 (0.3–4.8)

ER. PgR, HER2 *OR* Odd Ratio, *CI 95%* Confidence Interval 95%, * Statistic Signification <0.05

Regarding the association of the different immunohistochemical subtypes with the presence or not of HPV, the presence of HPV is related to the luminal B phenotypes (particularly HER2-negative), while in contrast the triple negative and luminal A phenotypes are more related to the absence of HPV. However, this relationship is not statistically significant (*p* = 0.055) (Table 5).

**Fig. 1:**
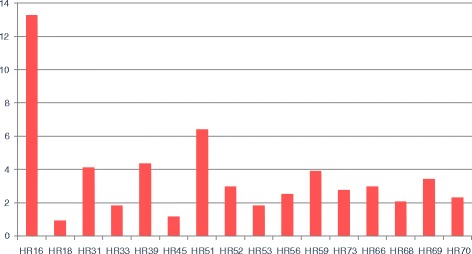
Percentage of high risk (HR) viral serotypes. Percentage of high risk (HR) viral serotypes with respect to total sample size

**Fig. 2:**
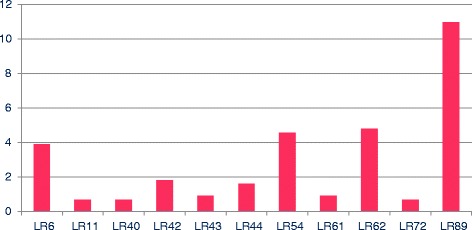
Percentage of low risk (LR) viral serotypes. Percentage of low risk (LR) viral serotypes with respect to total sample size

**Fig. 3:**
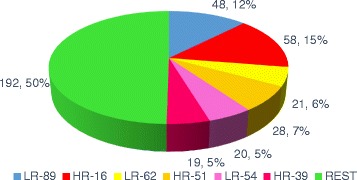
Proportion of viral serotypes found more frequently in this study

Within the global sample, 47% of the cases (*n* = 55) and 61.2% of the controls (*n* = 30) were infected by more than one HPV serotype. In other words, co-infection by more than one viral serotype was observed in 85 samples. With regard to the identified serotypes, in the 179 samples (130 cases and 49 controls) with the presence of viral DNA, we identified 16 different high risk serotypes and 11 low risk serotypes. Figures 1 and 2 show HPV serotype 16 to be the most frequent high risk serotype in both the cases and the controls, followed by HPV-89 (Fig. 3).

## Discussion

Band in 1991 [46] were the first to postulate that HPV might be implicated in breast cancer. These authors suggested that HPV-16/HPV-18 could immortalize the epithelial cells of normal mammary gland tissue through the inhibition of apoptosis. Shortly after, in 1992, Di Lonardo [47] by PCR techniques confirmed the presence of HPV-16 in 29.4% of 17 breast cancer samples supporting a potential relationship between HPV and breast carcinoma.

In the current study, the presence of HPV was shown in 51.8% of the cases and 26.3% of the controls. Of note, these results are higher than the prevalence described by some meta-analyses [30], in which HPV was found to be present in 23% of the cases and in 12.9% of the controls, although the difference here was also statistically significant. Nevertheless, it should be mentioned that there is a broad range of HPV-positive findings in breast cancer samples, depending on the geographical setting involved. In fact, according to Simoes [30], the prevalence in Europe is 13.4%, versus 42.9% in Australia and North America. The OR calculated by this author showed HPV-positive women to have a 5.9 fold higher risk of suffering breast cancer than HPV-negative women (95%CI: 3.36–10.67). The OR in our study was 4.034 (95% CI: 2.213–7.352), i.e., somewhat lower than in the above study.

Regarding the implications of the presence of HPV in benign disease (26.3% in this study), our hypothesis is that if we follow the pattern of cervical cancer and HPV, and if HPV is considered to be oncogenic for breast cancer, then it should be present both in this tissue and in some normal breasts or breasts with precancerous lesions (supposedly in lesser proportion).

In 2004, De Villiers [35] published the highest prevalence to date. She detected HPV in 86% of the cases (25/29 breast cancers) and in 69% of the nipple tissue samples of the same breasts used as controls (20/29). Other authors [38] have also used the same cancer-affected breast as control. However, in our opinion the use of these controls is questionable from a methodological perspective, since the breasts involved presented cancer and were, therefore, not normal. Most of the published studies do not follow a precise methodology, and the screening criteria used are very heterogeneous. Some studies only consider juvenile malignancies [34], while others include inflammatory breast cancer tissues [48, 49], triple-negative tumors [50], medullary malignancies [51], metaplastic breast cancer [52], papillary lesions [20, 53], Paget’s disease [54], or carcinoma in situ [9, 24, 25, 36, 41, 55–60]. In addition, no standards are used in selecting the molecular technique to screen for viruses, implying the potential detection of different viral serotypes. Therefore, it is quite likely that, discrepancies among the studies are due to the factors mentioned above.

The literature published to date describes the presence of both oncological high and low risk HPV serotypes, with a broad variety of HPV subtypes. Even cutaneous variants have been reported, as in the studies of De Villiers [61] or Ong [62], who found HPV-27 or −57, and HPV-4, respectively. Our data are consistent with the findings in the literature, according to which HPV-16 is the most frequently identified serotype. However, in our study a low risk serotype not previously reported was identified, and moreover was the most prevalent among all the cases: serotype HPV-89 (Fig. 2). A possible explanation for this observations is that we used different detection methods in order to increase the range of our findings.

On the other hand, in all published studies which include cases and controls, the prevalence of HPV has been found to be higher in the cases than in the controls. In contrast, Wang et al. [63] identified HPV in one sample of 7 breast cancers and in two benign disease samples. Obviously, this study presents clear limitations in terms of sample size. Most of the published articles lack a rigorous methodological design in relation to the calculation of sample size, a fact that can weaken the results obtained. In our study, the case and control groups were designed on a 1:1 basis, and although we finally included 251 cases and 186 controls (i.e., a precise 1:1 proportion was not achieved), the statistical power was maintained.

To our knowledge, our study includes the larger series of samples in which HPV has been analyzed by three different validated molecular methods. Recent studies by Li et al. [64] (including 187 breast cancers and 92 benign tumors) and by Fu et al. [43] (with 169 cases and 83 controls) both in China, have shown that HPV may have a possible causal role in breast cancer pathogenesis [65, 66] It could be concluded that demographic and genetic characteristics may be determinant in HPV-positive breast cancer, in view of the wide range of results obtained. This is a possible explanation because there is such a different prevalence.

In our study, the presence of HPV was associated (but not significant *p* value, *p* = 0.055) to luminal B-HER2-negative immunophenotype. This observation is consistent with high Ki-67 levels, since luminal B tumors present at least intermediate or high Ki-67 expression. In this respect, among HPV-positive tumors, about 40% were luminal B/HER2-negative. In this regard, El-Shinawi et al. [49] found the expression of Ki-67 significantly higher in both (inflammatory and non-inflammatory) breast cancer with viral DNAs. In contrast, Subhawong in 2009 [67] established a similarity between the immunophenotypic characteristics of triple-negative tumors (and more specifically of basal-like tumors) and HPV-positive squamous cell carcinomas (functional loss of the retinoblastoma tumor suppressor, presence of p16 or p53 overexpression). However, in that study of 33 triple-negative breast cancers, no viral DNA was identified by in situ hybridization techniques. Other authors [48, 50] have also reported significant differences with triple-negative tumors. Recently, in 2015, Fernandes et al. [60] found no statistically significant association between the molecular subtypes and the presence of HPV; however the sample size was very small (10 HPV-positive samples out of a total of 24).

It is well known that luminal B tumors are ER-dependent neoplasms, a condition which in turn favors the perpetuation of cervical HPV infection. Therefore, further studies are needed to confirm our results.

Emphasis should be placed on the importance of further studies to clarify the role of HPV in the carcinogenic mechanisms in breast cancer. First to clarify whether causal relationship between the virus and breast cancer actually exists. Human papillomavirus can be transmitted by skin-to-skin contact, as well as by sexual activity. Sexual transmission is the generally accepted transmission route, though it is not the only route, since transmission could occur by hand from the female perineum to the breast, wich could occur during sexual activity or even showering or bathing [8, 11, 68–70]. In an attempt to identify the possible origin of HPV in the breast, a number of authors [71–74] have explored the possible relationship between presence of the virus in the breast and cervical disease produced by HPV. Based on their studies it is not possible to conclude that HPV of the breast originates from the cervix. Further research is needed. On the other hand, De Villiers et al. [61] demonstrated the presence of HPV in 69% of the nipples of breasts with cancer. This as early as 2004 already suggested that HPV could gain access to the breast tissues through the nipple. Based on this idea, some investigators postulated breast milk as one of the main transmission routes of the virus, with the breast epithelial cells as the site of latent infection [9, 10].

Accordingly, breast epithelial cells that lose cell proliferation control are more susceptible to HPV infection. This loss of control is one of the first steps in breast carcinogenesis. Human papillomavirus infection in women takes place through contact by the hands or body fluids (e.g. breast milk...), with microfissures in the nipple serving as entry points for HPV. Errors may occur in the normal cell repair process, and this in combination with other cofactors can favour cell immortalization. Some of these immortal cells can be infected with viral DNA episomes or integrated DNA. The possible mechanisms whereby HPV intervenes in breast carcinogenesis may be the same as in the anogenital setting [42], through E6 and E7, though the viral load found in the breast is much lower.

The presence of HPV might also provide a new target allowing individualized patient treatment. The possibility of including antiviral agents as part of the strategy for the prevention (vaccines) [18, 75] and treatment of breast cancer could be a reality in the future, as it is currently done in other cancers, such as hepatocellular carcinoma or Kaposi’s sarcoma.

In contrast to other viruses with known neoplastic transformation potential, HPV can be defined as having “indirect” oncogenic capacity. The so-called “viral transforming genes”, which synthesize proteins involved in the inhibition and degradation of key mediators in cell division and the control of apoptosis (p53 and Rb), promote cellular susceptibility to neoplastic transformation due to the impossibility of repairing DNA errors induced by a series of intrinsic or extrinsic factors during cell division. The oncogenic action is therefore indirect, since there is no direct intervention as host gene promoters, regulators or inhibitors. Oncogenic papillomaviruses intervene in the cell division phase, promoting inhibition of the cellular repair capacity. This phenomenon, and the associated environmental circumstances, lead to the accumulation of errors, often acquired on a random basis (so called clastogenic effect), with a phenotype that is independent of the initial presence of the virus. No differences would therefore be expected in the phenotypic evolution of tumors induced by HPV or potentially induced by some other type of genetic-environmental event.

## Conclusions

In conclusion, this study of 251 cases and 186 controls has evidenced HPV DNA in 51.8% of the cases (breast cancer specimens) and in 26.3% of the controls (benign disease). Furthermore, the OR corresponding to HPV within the multivariate model, taking age and lactation into account, is 4.034. We have not been able to establish a significant relationship between the presence of viral DNA and the immunohistochemical subtypes. Nevertheless, there is a certain tendency to correlate the presence of HPV to the HER2- luminal B subtype (*p* = 0.055). In concordance with existing literature, the most prevalent serotype was found to be HPV-16. The strongly discrepant results in the literature are explained by the great methodological diversity found among the different studies. Our study, with methodological rigour and a sample size not previously found in the literature, demonstrate a significant presence of HPV DNA in the breast cancer samples. A possible causal relationship, or mediation or not as a cofactor, remains to be established by future studies.

## Abbreviations

CI: Confidence interval
ER: Estrogen receptor
FDA: Food and Drug Administration
FISH: Fluorescence in situ hybridization
HER2: Human epidermal growth factor receptor 2
HPV: Human papillomavirus
PCR: Polymerase chain reaction
PgR: Progesterone receptor
